# Supplementary material for: Orgo-Seq integrates single-cell and bulk transcriptomic data to identify cell type specific-driver genes associated with autism spectrum disorder
Source: Nat Commun. 2022 Jun 10;13:3243. doi: 10.1038/s41467-022-30968-3 (PMC9187732; doi:10.1038/s41467-022-30968-3)
Supplement: Supplementary file 1 — Supplementary Information [file 41467_2022_30968_MOESM1_ESM.pdf]

SUPPLEMENTARY FIGURES

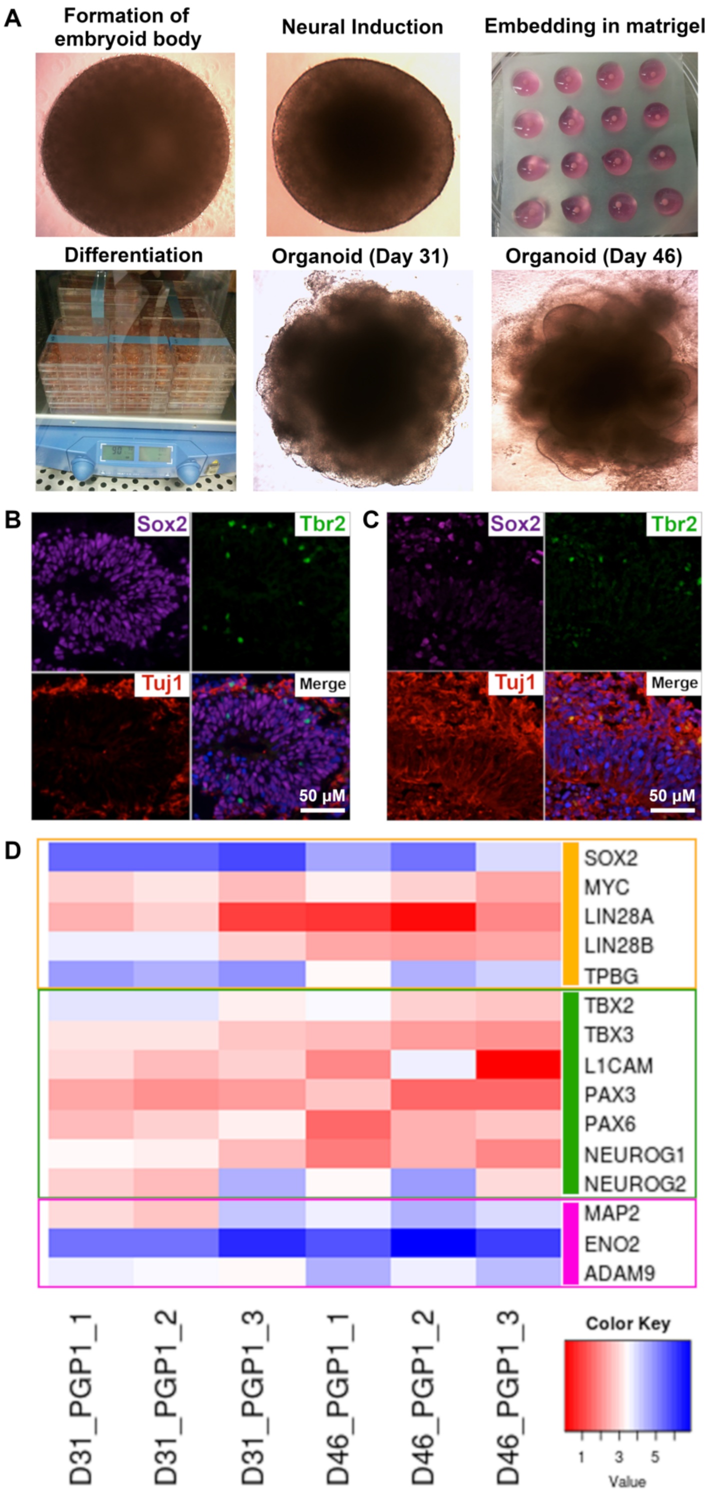

Supplementary Figure 1. Characterization of cerebral organoids. (A) As previously described in Lancaster et al., 2014, we started with the formation of an embryoid body, followed by neural induction, embedding in matrigel and differentiation on an orbital shaker. (B) Markers for an organoid at Day 31 are shown, with 3 independent sections of an organoid showing similar results. (C) Markers for an organoid at Day 46 are shown, with 3 independent sections of an organoid showing similar results. (D) Transcriptomic profiling using RNA sequencing showed a similar pattern across stem cell markers (in orange box), neural progenitor cell markers (in green box) and neuronal cell markers (in pink box) for organoids differentiated for 31 days (D31_PGP1_1, D31_PGP1_2 and D31_PGP1_3) compared to organoids differentiated for 46 days (D46_PGP1_1, D46_PGP1_2 and D46_PGP1_3). The experiments were repeated independently with 3 replicates for each timepoint. The values represented by the heatmap shows the $\log_2$(FPKM) values.

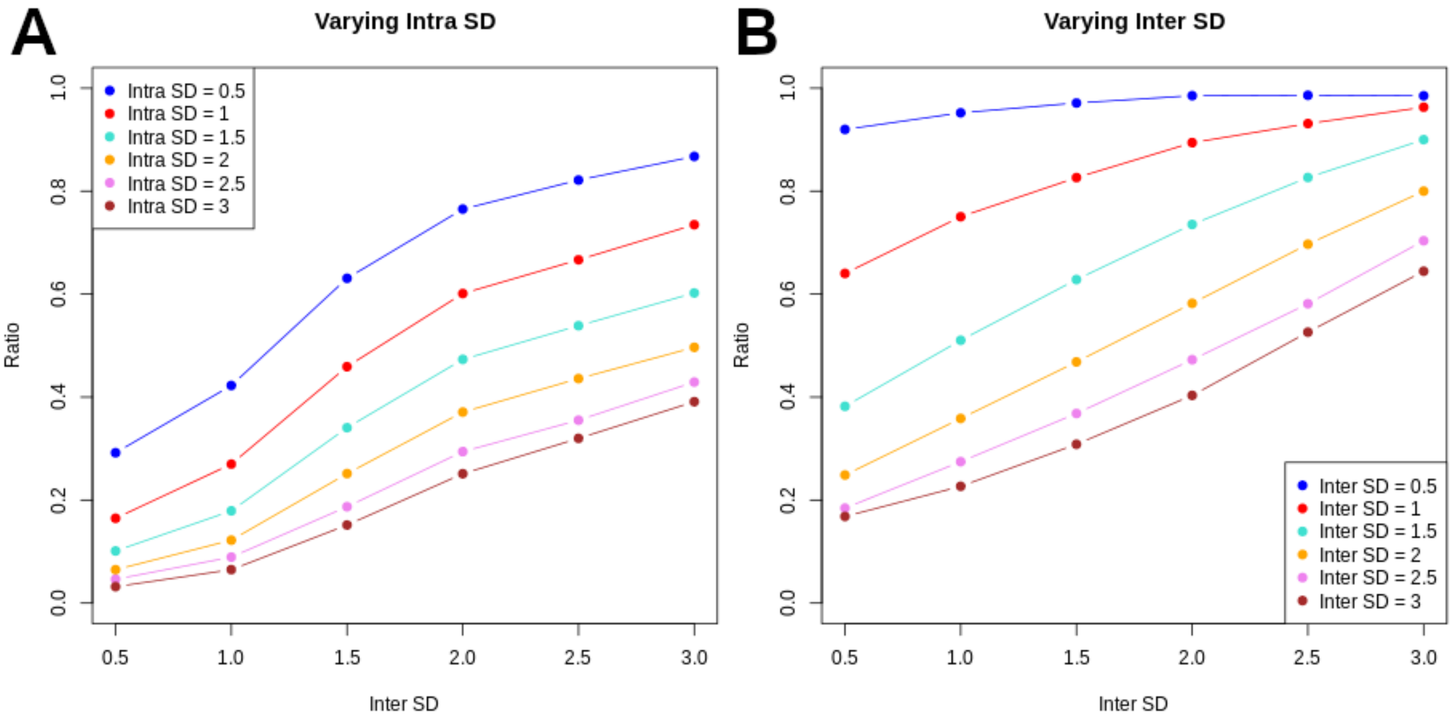

Supplementary Figure 2. Varying intra-individual and inter-individual standard deviations (SD). (A) The ratios of overlap between the genes with high intra-individual SDs and high inter-individual SDs, with intra-individual SDs varying from 0.5 to 3. (B) The ratios of overlap between the genes with high inter-individual SDs and high intra-individual SDs, with inter-individual SDs varying from 0.5 to 3.

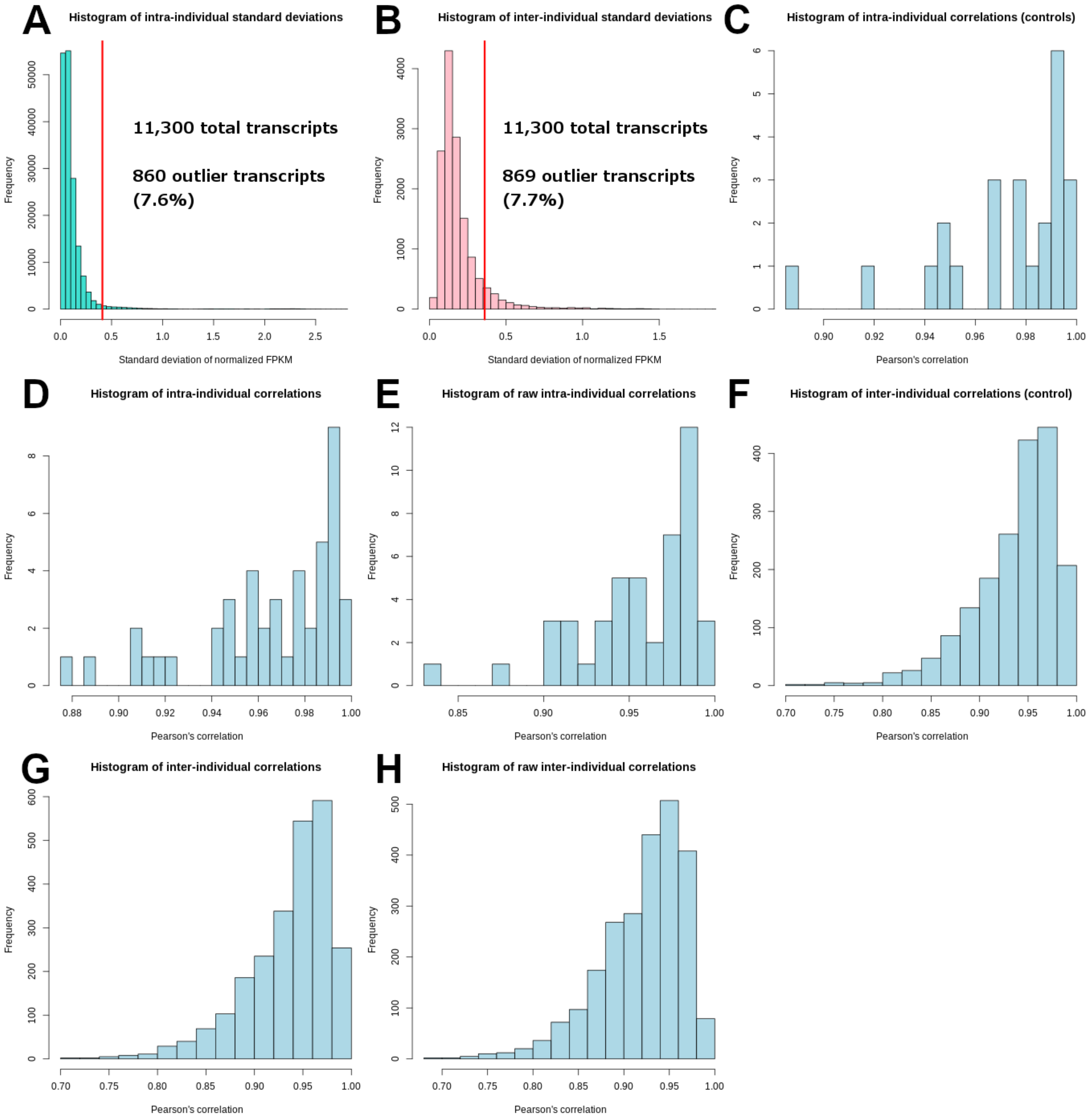

Supplementary Figure 3. Intra-individual and inter-individual correlations. (A) Standard deviations of normalized expression across replicates for each individual. (B) Standard deviations of normalized expression across replicates from different individuals. (C) Intra-individual Pearson's correlations for control individuals after removing outlier genes. (D) Intra-individual Pearson's correlations for all individuals after removing outlier genes. (E) Intra-individual Pearson's correlations r^2 for all individuals prior to removing outlier genes. (F) Inter-individual Pearson's correlations r^2 for control individuals after removing outlier genes. (G) Histogram showing the inter-individual Pearson's correlations r^2 for all individuals after removing outlier genes. (H) Histogram showing the inter-individual Pearson's correlations r^2 for all individuals prior to removing outlier genes.

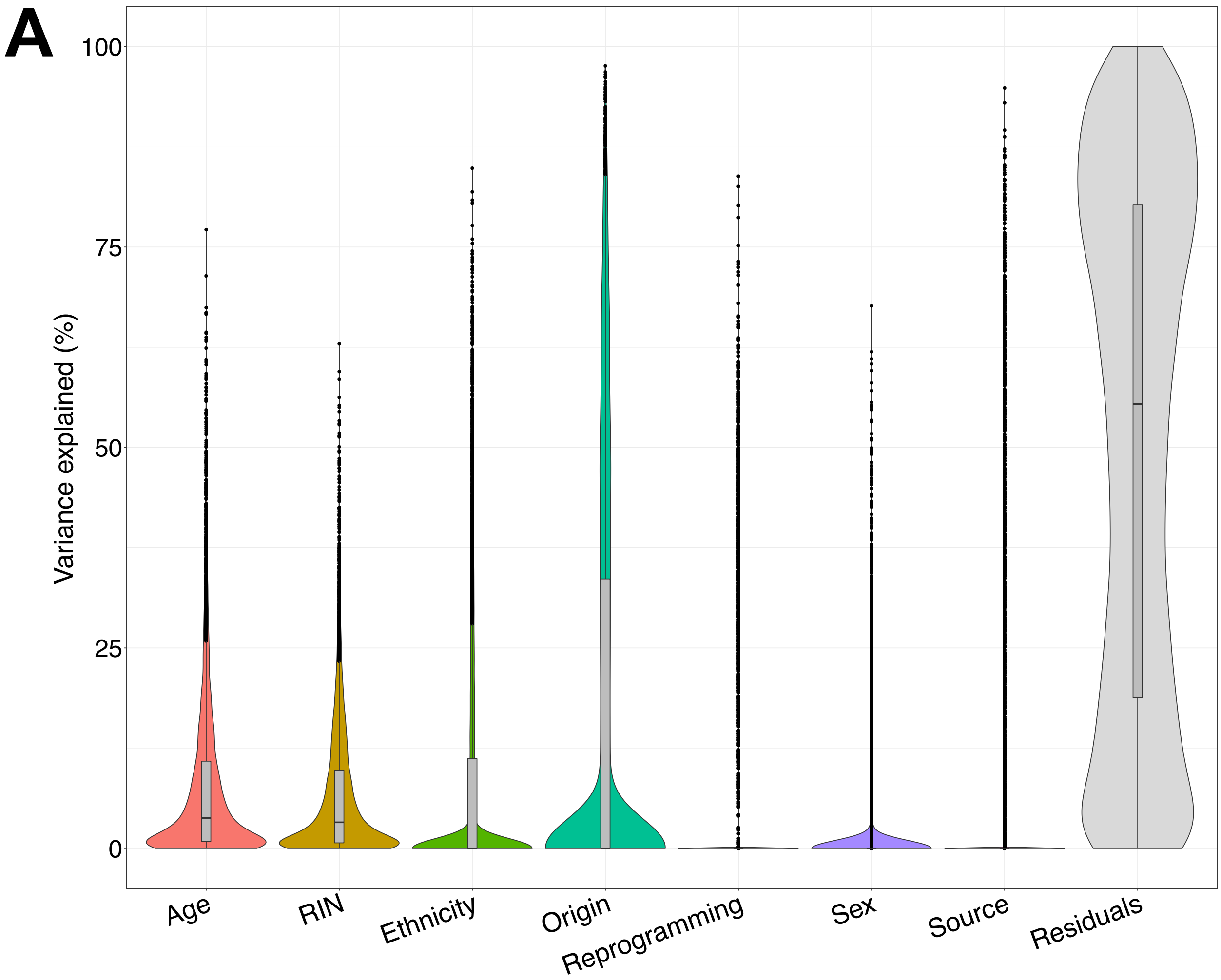

B

PANTHER GO	Observed	Expected	Fold Enrichment	Raw P-value	FDR
G protein-coupled receptor signaling pathway	9	29.83	0.30	1.17E-05	1.66E-02
organelle organization	119	81.26	1.46	1.77E-05	1.85E-02
cellular localization	88	56.61	1.55	4.65E-05	3.31E-02
detection of chemical stimulus involved in sensory perception of smell	0	10.82	<0.01	4.61E-05	3.44E-02
defense response	13	34.32	0.38	4.24E-05	3.50E-02
protein ubiquitination	36	16.95	2.12	5.48E-04	3.74E-02

Supplementary Figure 4. variancePartition analyses. (A) Percentage of variance in gene expression using 1 replicate from each of the 12 control donors, with the variance explained (*y*-axis) by 7 factors (age of donor, RIN values of samples, ethnicity of donor, tissue of origin, type of reprogramming, sex of donor and repository source of iPSCs) and the residuals (*x*-axis). Data in the boxes in violin plots are presented as median values with 25 percentile (lower bound of box) and 75 percentile (upper bound of box), and the points represent the maximum values, and 0 is the minimum value. (B) Gene ontology enrichment analyses for genes with >95% of the variance explained by the residuals. The *P*-values are calculated using two-sided Fisher's Exact Test with FDR adjustment for multiple comparisons.

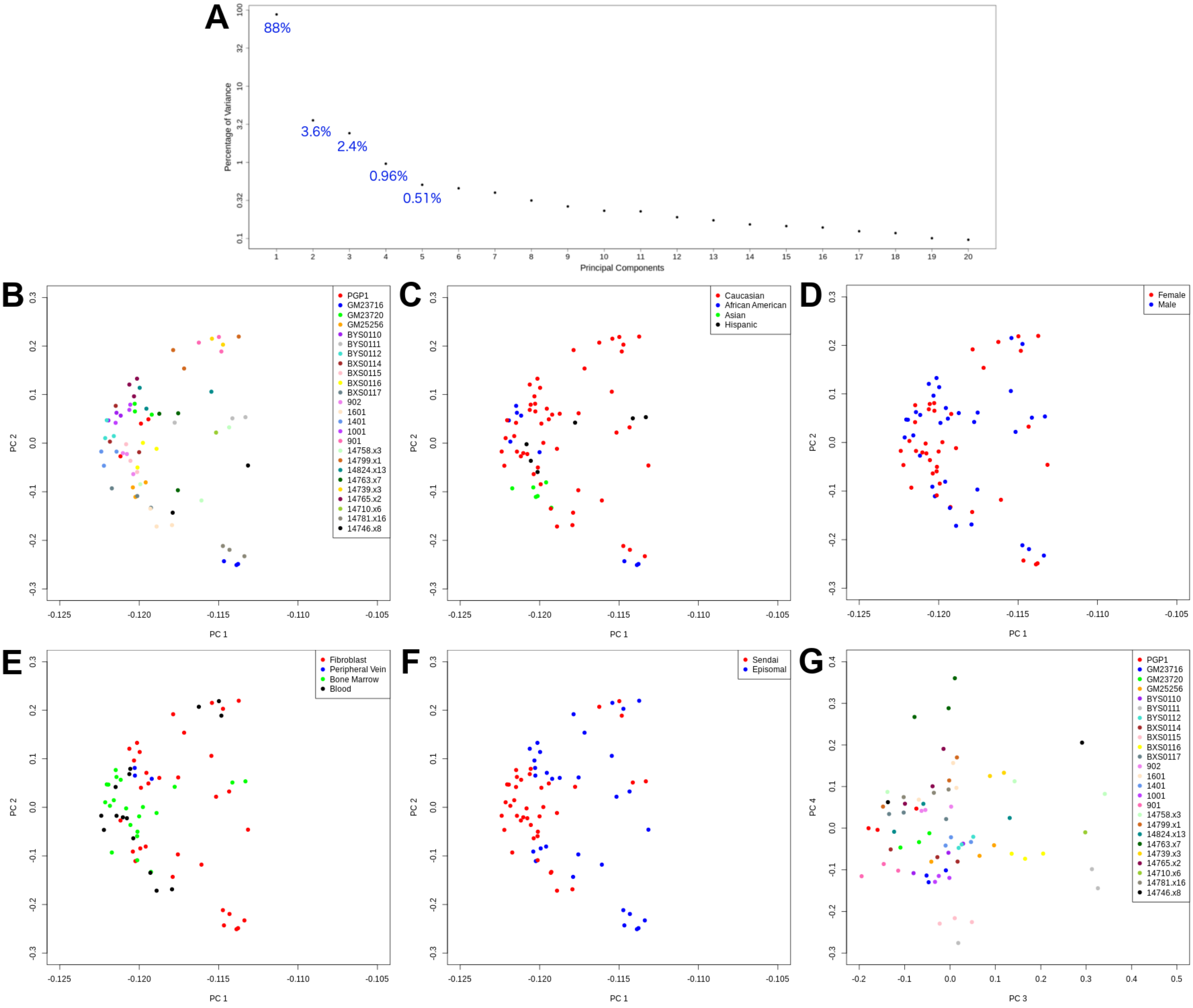

Supplementary Figure 5. Principal components analyses. (A) Variance in gene expression (y-axis) accounted for by the first 20 principal components (x-axis). (B) First two principal components (PC1 and PC2) showing classified by sample replicates. (C) First two principal components (PC1 and PC2) classified by ethnicity. (D) First two principal components (PC1 and PC2) classified by sex. (E) First two principal components (PC1 and PC2) classified by the origin of reprogramming. (F) First two principal components (PC1 and PC2) classified by the type of reprogramming. (G) Principal component 3 (PC3) and principal component 4 (PC4) classified by sample replicates.

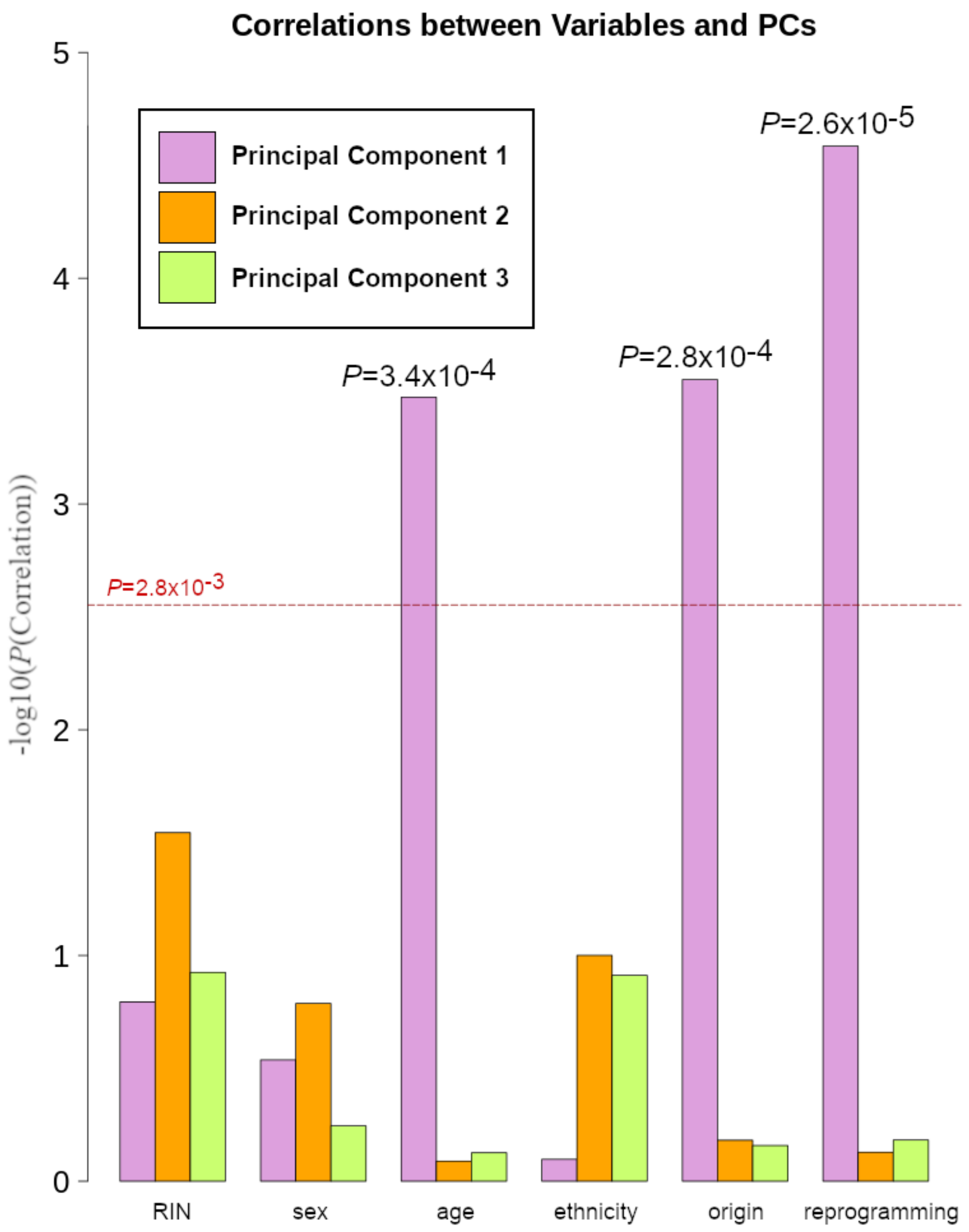

Supplementary Figure 6. Correlation of sample variables with principal components (PCs). Two-sided correlation tests using Pearson's correlation were performed between each sample variable (RIN, Sex, Age, Ethnicity, Origin of tissue, and Reprogramming method), and the $-\log_{10}$(P(Correlation)) values were plotted for the principal component 1 (PC1) in pink, the principal component 2 (PC2) in orange, and the principal component 3 (PC3) in green. The red dotted line indicates the significance threshold after Bonferroni correction ($P=2.8\times10^{-3}$) for multiple comparisons. These results show that sample variables such as age of the individual, origin of sample and type of reprogramming are highly correlated and are captured by PC1.

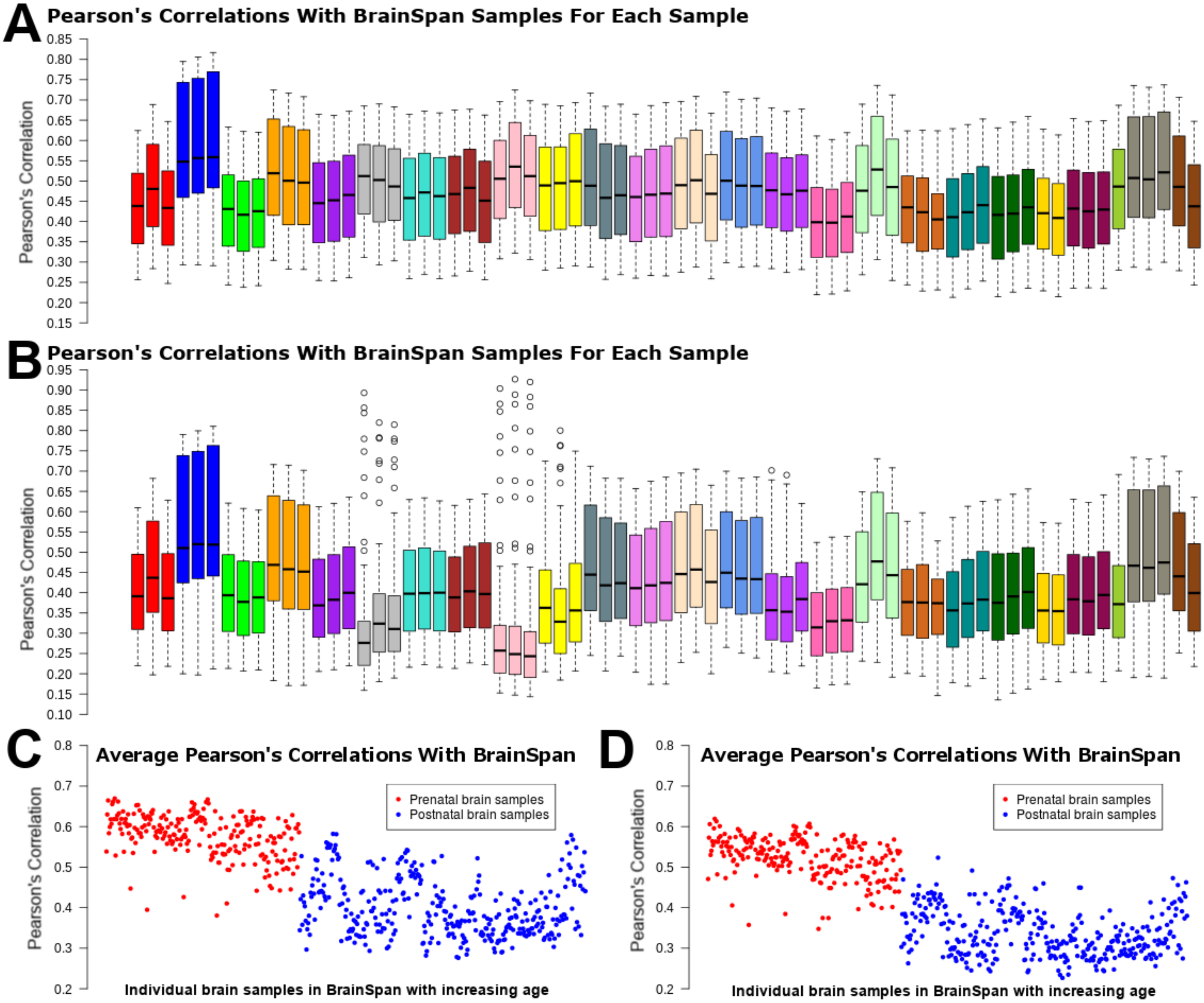

Supplementary Figure 7. Correlation with RNA sequence data from the cerebral organoids and post-mortem brain samples from the BrainSpan Project. Data in the boxes in boxplots are presented as median values with 25 percentile (lower bound of box) and 75 percentile (upper bound of box), and the whiskers/points represent the minimum or maximum values. (A) After removing outlier genes, correlations between the transcriptome data from cerebral organoids with post-mortem brain samples, for each organoid sample (x-axis). Each boxplot represents data from a single replicate, and replicates from the same donor are highlighted with the same color. The first 12 donors are control donors (3 replicates per donor), and the next 23 donors are cases with 16p11.2 deletions or 15q11-13 duplications (1-3 replicates per donor). (B) Prior to removing outlier genes, correlations between the transcriptome data from cerebral organoids with post-mortem brain samples, for each organoid sample (x-axis). Each boxplot represents data from a single replicate, and replicates from the same donor are highlighted with the same color. The first 12 donors are control donors, and the next 23 donors are cases with 16p11.2 deletions or 15q11-13 duplications. (C) After removing outlier genes, mean correlations between transcriptome data from cerebral organoids with individual prenatal brain samples (red), compared to postnatal brain samples (blue). (D) Prior to removing outlier genes, mean correlations between transcriptome data from cerebral organoids with individual prenatal brain samples (red), compared to postnatal brain samples (blue).

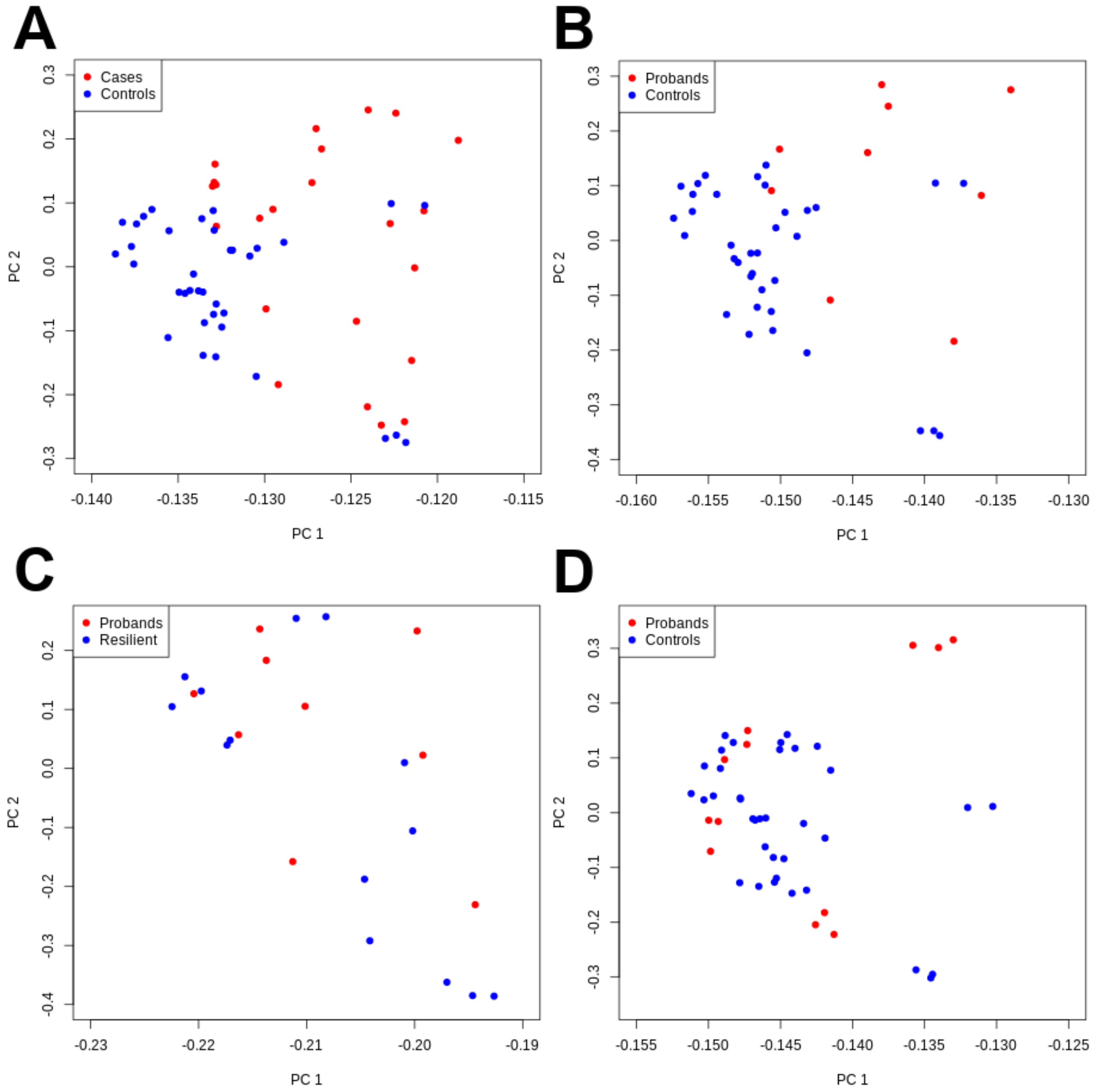

Supplementary Figure 8. Plots for the first two principal components in the differential expression analyses. (A) First 2 principal components (PC1 and PC2) for the samples from the cases with 16p11.2 deletions versus unaffected controls without 16p11.2 deletions (SetA). (B) First 2 principal components (PC1 and PC2) for the samples from the probands with ASD and 16p11.2 deletions versus unaffected controls without 16p11.2 deletions (SetP). (C) First 2 principal components (PC1 and PC2) for the samples from the probands with 16p11.2 deletions versus resilient individuals with 16p11.2 deletions (SetD). (D) First 2 principal components (PC2 and PC3) for the samples from the probands with ASD and 15q11-13 duplications versus unaffected controls.

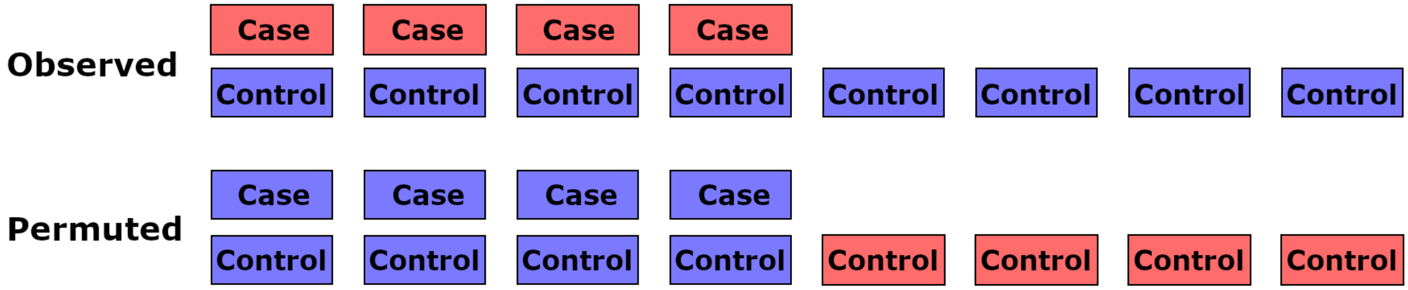

Supplementary Figure 9. Schematic of our permutation design. The schematic of our permutation design, where the observed case samples are colored in red, while the observed control samples are colored in blue. We sampled the number of observed cases from the observed controls only, and assigned all initial observed cases as "pseudo-controls" in the permutations. In the figure, we have 4 observed case samples and 8 observed control samples that we calculated the observed CellScores and GeneScores from. In the permutations, we sampled 4 "pseudo-cases" from the control samples randomly and assigned these "pseudo-cases" to be cases in the permutations (blue-colored cases). The remaining 4 control samples (blue-colored controls), as well as the observed case samples (red-colored controls), are then assigned to be "pseudo-controls" in the permutation. Using this approach, we ensure that we do not result in a scenario where one of the permutations is exactly the same as the observation.
