# Supplementary material for: Orgo-Seq integrates single-cell and bulk transcriptomic data to identify cell type specific-driver genes associated with autism spectrum disorder
Source: Nat Commun. 2022 Jun 10;13:3243. doi: 10.1038/s41467-022-30968-3 (PMC9187732; doi:10.1038/s41467-022-30968-3)
Supplement: Supplementary file 3 — Description of Additional Supplementary Files [file 41467_2022_30968_MOESM3_ESM.pdf]

Description of Additional Supplementary Files

Supplementary Data 1: DNA Integrity Numbers (DINs) and RNA Integrity Numbers (RINs) for all samples across all individuals.
The DIN values for DNA extracted from donor iPSC lines, and RIN values for RNA from the organoids.

Supplementary Data 2: Array CGH results for all iPSCs.
The CNVs detected from all iPSCs using array CGH.

Supplementary Data 3: CNVs detected from whole-exome sequence data from iPSC lines.
The CNVs detected using CoNIFER from whole-exome sequence data.

Supplementary Data 4: CNVs detected from whole-genome sequence data from iPSC lines.
The CNVs detected using CNVnator from whole-genome sequence data.

Supplementary Data 5: Variability and gene ontology analyses for highly variable genes.
The intra-individual and inter-individual standard deviation (SD) analyses, highly variable outlier genes and gene ontology results for the highly variable genes. The *P*-values in the gene ontology results are calculated using two-sided Fisher's Exact Test with FDR adjustment for multiples comparisons.

Supplementary Data 6: Power calculations for SetD.
The power calculations for SetD comparisons between individuals with ASD and 16p11.2 deletions versus resilient individuals with 16p11.2 deletions.

Supplementary Data 7: Cell type specific and non-cell type specific genes.
Genes found to be uniquely expressed in each cell cluster were labeled as "cell type specific genes", while genes found to be expressed in multiple cell clusters were labeled as "non-cell type specific genes".

Supplementary Data 8: CellScores and *P*(CellScores) for the 16p11.2 and 15q11-13 loci.
The critical cell cluster results for the 16p11.2 (SetA) and 15q11-13 data are shown.

Supplementary Data 9: Percentage overlaps among the 24 cell type clusters in the Tanaka study.
Pairwise calculations of the number of genes that are found in common between 2 cell type clusters from the Tanaka study, as well as the percentage overlap. Clusters with high percentage overlaps across multiple cell type clusters are discarded from the analyses.

Supplementary Data 10: Fine-mapping of cell type clusters onto neurodevelopmental maps.
The mean percentage overlaps among the genes from the cell type clusters identified in the Quadrato and Tanaka studies with the clusters from the neurodevelopmental map 1 (Velasco *et al.*) and neurodevelopmental map 2 (Eze UC *et al.*).

Supplementary Data 11: Cross-comparisons of cell type clusters from the Quadrato and Tanaka scRNA-seq studies.
Pairwise calculations of the Pearson's correlations (r) and *P*-values calculated using two-sided t-test for each cell type cluster between the Quadrato and Tanaka studies across both neurodevelopmental maps.

Supplementary Data 12: Critical cell types identified from cerebral organoids and post-mortem brain samples with 15q11-13 duplications.
The critical cell type results for the 15q11-13 locus are shown using post-mortem brain samples (cortex) with 15q11-13 duplications.

Supplementary Data 13: Percentiles of gene expressions for the genes in the 16p11.2 and 15q11-13 loci.
The mean expression of the genes for both the 16p11.2 and 15q11-13 loci.

Supplementary Data 14: GeneScores and *P*(GeneScores) for the top 2 critical cell types for the 16p11.2 locus and the top critical cell type for the 15q11-13 locus.
The driver gene results for the 16p11.2 (SetA) and 15q11-13 data are shown.

Supplementary Data 15: Full set of cell type specific GeneScores and *P*(GeneScores) for the 16p11.2 and 15q11-13 loci.
The full set of driver gene results for the 16p11.2 (SetA) and 15q11-13 data are shown.

Supplementary Data 16: Differentially expressed genes in common between 16p11.2 and 15q11-13.
The differentially expressed genes that are found in common between 16p11.2 (SetA) and 15q11-13, as well as 16p11.2 (SetP) and 15q11-13.
