# Supplementary material for: Orgo-Seq integrates single-cell and bulk transcriptomic data to identify cell type specific-driver genes associated with autism spectrum disorder
Source: Nat Commun. 2022 Jun 10;13:3243. doi: 10.1038/s41467-022-30968-3 (PMC9187732; doi:10.1038/s41467-022-30968-3)
Supplement: Supplementary file 20 — Reporting Summary [file 41467_2022_30968_MOESM20_ESM.pdf]

nature portfolio

Corresponding author(s): Elaine T. Lim, George M. Church

Last updated by author(s): Apr 17, 2022

Reporting Summary

Nature Portfolio wishes to improve the reproducibility of the work that we publish. This form provides structure for consistency and transparency in reporting. For further information on Nature Portfolio policies, see our Editorial Policies and the Editorial Policy Checklist.

Statistics

For all statistical analyses, confirm that the following items are present in the figure legend, table legend, main text, or Methods section.

n/a	Confirmed	
☐	☒	The exact sample size (*n*) for each experimental group/condition, given as a discrete number and unit of measurement
☐	☒	A statement on whether measurements were taken from distinct samples or whether the same sample was measured repeatedly
☐	☒	The statistical test(s) used AND whether they are one- or two-sided<br>*Only common tests should be described solely by name; describe more complex techniques in the Methods section.*
☐	☒	A description of all covariates tested
☐	☒	A description of any assumptions or corrections, such as tests of normality and adjustment for multiple comparisons
☐	☒	A full description of the statistical parameters including central tendency (e.g. means) or other basic estimates (e.g. regression coefficient) AND variation (e.g. standard deviation) or associated estimates of uncertainty (e.g. confidence intervals)
☐	☒	For null hypothesis testing, the test statistic (e.g. *F*, *t*, *r*) with confidence intervals, effect sizes, degrees of freedom and *P* value noted<br>*Give P values as exact values whenever suitable.*
☒	☐	For Bayesian analysis, information on the choice of priors and Markov chain Monte Carlo settings
☒	☐	For hierarchical and complex designs, identification of the appropriate level for tests and full reporting of outcomes
☐	☒	Estimates of effect sizes (e.g. Cohen's *d*, Pearson's *r*), indicating how they were calculated

Our web collection on statistics for biologists contains articles on many of the points above.

Software and code

Policy information about availability of computer code

Data collection	Reads were trimmed using Trimmomatic v0.32 (Bolger A et al., Bioinformatics 2014), then mapped to the hg19 human genome sequence using TopHat v2.0.13 (Kim D et al., Genome Biol 2013), and transcript assembly was performed using Cufflinks v2.2.1 (Trapnell C et al., Nat Biotechnol 2010) to calculate the fragments per kilobase per million reads (FPKM) values for each transcript. In addition, the reads were mapped to the hg19 sequence using STAR v2.4.0f1 (Dobin A et al., Bioinformatics 2013), and single nucleotide variant calling on the aligned sequences was performed using GATK v3.3-0 HaplotypeCaller (McKenna A et al., Genome Res 2010). CNV calling was performed using CoNIFER v0.2.2 (Krumm N et al., Genome Res 2012) and CNVnator v0.4.1 (Abyzov A et al., Genome Res 2011).
Data analysis	The custom codes used in our data analysis are available at: https://gitlab.com/elimlab/orgo-seq

For manuscripts utilizing custom algorithms or software that are central to the research but not yet described in published literature, software must be made available to editors and reviewers. We strongly encourage code deposition in a community repository (e.g. GitHub). See the Nature Portfolio guidelines for submitting code & software for further information.

Data

Policy information about availability of data

All manuscripts must include a data availability statement. This statement should provide the following information, where applicable:

- Accession codes, unique identifiers, or web links for publicly available datasets
- A description of any restrictions on data availability
- For clinical datasets or third party data, please ensure that the statement adheres to our policy

CNVs were compared to the Database of Genomic Variants CNV-DGV_hg19_May2016 (http://dgv.tcag.ca/dgv/app/home) to identify CNVs that are common in the general population. Annotation for the single nucleotide variants was performed using SeattleSeq Annotation 138 (https://snp.gs.washington.edu/

SeattleSeqAnnotation138/). The raw data generated by our study has been uploaded to SRA (acc code: PRJNA824347) and the processed data has been uploaded to GEO (acc code: GSE200851).

Field-specific reporting

Please select the one below that is the best fit for your research. If you are not sure, read the appropriate sections before making your selection.

- [x] Life sciences
- [ ] Behavioural & social sciences
- [ ] Ecological, evolutionary & environmental sciences

For a reference copy of the document with all sections, see nature.com/documents/nr-reporting-summary-flat.pdf

Life sciences study design

All studies must disclose on these points even when the disclosure is negative.

Sample size	We used the largest number of induced pluripotent stem cells available to us in this current study. We had also performed RNA sequencing on as many samples as available.
Data exclusions	Transcripts with no expression or outlier variances were excluded from the analyses and the exclusion criteria have been described in the manuscript.
Replication	For each donor, we differentiated a single cerebral organoid in a well, and pooled 20 organoids from the same donor across different wells and plates to obtain a replicate sample for RNA sequencing.
Randomization	We compared the RNA sequence data from individuals (affected or unaffected) with 15q11-13 duplications or 16p11.2 deletions with unaffected individuals without the duplications or deletions. The first principle component was included as a co-variate.
Blinding	Blinding was not relevant to our study as we confirmed the genotype status for each donor (absence or presence of 16p11.2 deletion or 15q11-13 duplication).

Reporting for specific materials, systems and methods

We require information from authors about some types of materials, experimental systems and methods used in many studies. Here, indicate whether each material, system or method listed is relevant to your study. If you are not sure if a list item applies to your research, read the appropriate section before selecting a response.

Materials & experimental systems

n/a	Involved in the study	
[ ]	[x]	Antibodies
[ ]	[x]	Eukaryotic cell lines
[x]	[ ]	Palaeontology and archaeology
[x]	[ ]	Animals and other organisms
[x]	[ ]	Human research participants
[x]	[ ]	Clinical data
[x]	[ ]	Dual use research of concern

Methods

n/a	Involved in the study	
[x]	[ ]	ChIP-seq
[x]	[ ]	Flow cytometry
[x]	[ ]	MRI-based neuroimaging

Antibodies

Antibodies used	Cryosections of fixed cerebral organoids were immunostained with antibodies against Sox2 (Santa Cruz sc-17320, 1:200 dilution), Tbr2 (Abcam ab-23345, 1:200 dilution), Tuj1 (Covance MMS-435P, 1:1000 dilution) and Alexa Fluor secondary antibodies (ThermoFisher).
Validation	Primary antibodies used in this study were validated for homo sapiens in several previous publications, such as Sox2 (Pan H et al., Cell Res 2016), Tbr2 (Eze UC et al., Nat Neurosci 2021) and Tuj1 (Savvaki M et al., EJN 2021).

Eukaryotic cell lines

Policy information about cell lines

Cell line source(s)	The sources of the induced pluripotent stem cell lines used to differentiate cerebral organoids are described in Table 1. HEK293T cells were kindly given by Professor Vijaya Ramesh's lab in the Center for Genomic Medicine at Massachusetts General Hospital. The GM8330 induced pluripotent stem cell line used to differentiate neural progenitor cells and induced neurons were previously provided by Professor Stephen Haggarty's lab at Harvard University (Sugathan A et al., PNAS 2014).
Authentication	We checked the single nucleotide variants detected from RNA sequencing for replicates from the same induced pluripotent

	stem cell, to ensure that there is no mix-up between the replicates from the same individual. In addition, we compared the concordance of the SNPs called from DNA whole-exome sequence data with SNPs called from RNA sequence data from all individuals to ensure that there are no mix-ups in the samples. Array CGH data was also generated from each individual iPSC and compared with the genotype information for each individual. The HEK293T cells were authenticated in prior work in Professor Vijaya Ramesh's lab (Han S et al., JBC 2012). The authentication for the GM8330 induced pluripotent stem cell line was previously performed in prior work in the Talkowski lab (Sugathan A et al., PNAS 2014).
Mycoplasma contamination	All iPSCs and cerebral organoids were tested negative for mycoplasma using the LookOut Mycoplasma PCR Detection kit (Sigma MP0035).
Commonly misidentified lines (See ICLAC register)	None.
